# Gorgas' Dental Medicine

**Published:** 1884-05

**Authors:** 


GORGAS' DENTAL MEDICINE.—From *The Dental Cosmos*, Philadelphia, March, 1884 :—

" The author of the volume before us, a distinguished teacher and writer, is well and favorably known to his profession. * * * The work may be said to be the first elaborate attempt to supply a want which every practitioner of Dentistry has experienced. It contains a large amount of useful information which is not to be found in any other volume.

From Prof. Frank Abbott, M. D., D. D. S., New York College of Dentistry :—

" I am so pleased with the work that I shall, with pleasure, recommend it to the students of our College (as well as to the profession generally), and will add it to the list of text-books."

From Prof. J. S. B. Alleyne, Missouri Dental College, St. Louis :—

" Thoroughly correct, and admirably adapted to the wants of the dental student."

From *The Independent Practitioner*, Buffalo, New York, March 1884 :—

" We most heartily recommend it to every one who is desirous of a complete text-book upon dental therapeutics."

Prof. Jonathan Taft, in the *Dental Register*, February, 1884.

" Perhaps no one in this country is better able to prepare such a manual than Prof. Gorgas. He has large experience in various directions, that has, in an eminent degree, qualified him for such a task."

C. T. Stockwell, D. D. S., Editor of the *New England Journal of Dentistry* :—

" I have no criticisms to make of the work. It appears to me to be most admirable for the purpose for which it is evidently intended."

From the *Dental Jairus*, San Francisco, February, 1884.

" No man in the dental profession is better qualified to prepare such a book. * * * The great number of Tables and Dental Formulæ which it contains makes it truly valuable.

From the *Dental Practitioner*, Philadelphia, March, 1884.

" The best book of the kind with which the dental profession has been favored. * * * We have not attempted to enumerate *all* the good things in this book, but sufficient to indicate its general character. The need of such a work has long been felt by the profession, and we make no doubt its rapid sale will express their approval of it."

From the *Dental News*, Indiana, February, 1884.

" We have never examined a work, pertaining to dental science that we can more heartily endorse for real practical merit than this.

